# A Neurologist's Guide to REM Sleep Behavior Disorder

**DOI:** 10.3389/fneur.2020.00610

**Published:** 2020-07-08

**Authors:** Amber Roguski, Dane Rayment, Alan L. Whone, Matt W. Jones, Michal Rolinski

**Affiliations:** ^1^School of Physiology, Pharmacology and Neuroscience, University of Bristol, Bristol, United Kingdom; ^2^Rosa Burden Centre, Southmead Hospital, Bristol, United Kingdom; ^3^Department of Neurology, Southmead Hospital, Bristol, United Kingdom; ^4^Translational Health Sciences, University of Bristol, Bristol, United Kingdom

**Keywords:** REMsleep behavior disorder (RBD), Parkinson's disease, prodromal Parkinson's disease, sleep disorders, neurology, neuroscience, sleep

## Abstract

REM Sleep Behavior Disorder (RBD) is a chronic sleep condition characterized by dream enactment and loss of REM atonia. Individuals often present to clinic with complaints of injury to themselves or their bed-partner due to violent movements during sleep. RBD patients have a high risk of developing one of the neurodegenerative α-synucleinopathy diseases: over 70% will develop parkinsonism or dementia within 12 years of their diagnosis. RBD patients also exhibit accelerated disease progression and a more severe phenotype than α-synucleinopathy sufferers without RBD. The disease's low prevalence and the relatively limited awareness of the condition amongst medical professionals makes the diagnosis and treatment of RBD challenging. Uncertainty in patient management is further exacerbated by a lack of clinical guidelines for RBD patient care. There are no binary prognostic markers for RBD disease course and there are no clinical guidelines for neurodegeneration scaling or tracking in these patients. Both clinicians and patients are therefore forced to deal with uncertain outcomes. In this review, we summarize RBD pathology and differential diagnoses, diagnostic, and treatment guidelines as well as prognostic recommendations with a look to current research in the scientific field. We aim to raise awareness and develop a framework for best practice for RBD patient management.

## Symptoms and Diagnostic Considerations

Rapid Eye Movement (REM) Sleep Behavior Disorder (RBD) is a non-familial sleep disorder, characterized by the loss of the inherent muscle atonia observed during normal REM sleep. This phenomenon is often referred to as REM Sleep without Atonia (RSWA). Whilst isolated RSWA is frequently an incidental finding in sleep studies, it forms the substrate of the dream enactment behavior which defines RBD. Here, individuals experience vivid dreams which they act out during sleep.

It is important to remember that dream enactment and limb movements during sleep can occur in the healthy population, often in the context of heightened emotional states (1–3). The same symptoms may also be experienced during withdrawal from sedatives or alcohol. In non-pathological dream enactment, individuals typically respond to dream content during the transition from REM sleep to the awake state and while maintaining REM atonia during much of the REM period. In contrast, RBD individuals will maintain REM sleep during and immediately after most of their dream enactments. As acute dream enactment is generally self-limiting, the chronicity of symptoms (>6 months) is a key distinguishing factor, and forms part of the diagnostic criteria for RBD (4).

Anecdotally, dreams are often reported by patients with RBD as violent or aggressive, resulting in violent motor behaviors which may pose a threat to them or their bedpartner (5). Whilst accounts of individuals kicking, punching, biting, or even strangling their bedpartners during sleep paint an emotive image of the condition and often capture public interest, they are prone to recall bias. More systematic studies have revealed that violent dreams and behaviors only make up a small percentage of all events (6–8). When a dream enactment is occurring, the individual's eyes will remain closed as they engage with the dream environment and their movements are generally contained to their immediate surroundings, thus differentiating these episodes from NREM parasomnias such as sleepwalking (4). Upon awakening from a large motor event, the RBD individual will be alert and orientated to their surroundings (4).

The frequency of motor events may vary greatly between RBD individuals; ranging from multiple episodes per night, to one episode per month (9). In any one patient, the severity and frequency of the behaviors may also vary from night to night, and over the course of their condition (10). The mechanisms behind this fluctuation remains unknown.

### Etiology

The behavioral states of wake and sleep are initiated and maintained by complex interplay between multiple brainstem and diencephalic nuclei. Dysregulation, disease or degeneration of these nuclei can result in sleep disorders, such as narcolepsy, and subtle changes to sleep-wake patterns. In the case of RBD, the primary pathology appears to be an excitation/inhibition imbalance in the brainstem nuclei controlling REM muscle tone.

Movement during REM sleep is controlled by two systems: one controls the input to spinal cord motoneurons to generate muscle atonia (extrapyramidal), and the other controls motor cortex activation to suppress locomotor activity (pyramidal). The main generator of REM-sleep is the predominantly-glutamatergic Subcoeruleus/Pre-Locus Coeruleus complex [SubC/PC- analogous to the rat/mouse sublaterodorsal nucleus (SLD)], which is anatomically situated just below the noradrenergic locus coeruleus in the pons (11). As well as projecting to many subcortical brain regions to promote and maintain REM sleep, the SubC/PC projects caudally to control the REM atonia neural network (12). Preceding and during REM sleep, the REM-active SubC/PC excites the inhibitory ventromedial medulla (VMM) and glycinergic neurons of the spinal ventral horn, which in turn tonically hyperpolarize spinal motor neurons (12, 13). This results in a temporary paralysis of skeletal muscles and thus significantly reduced REM muscle tone.

**Figure 1:**
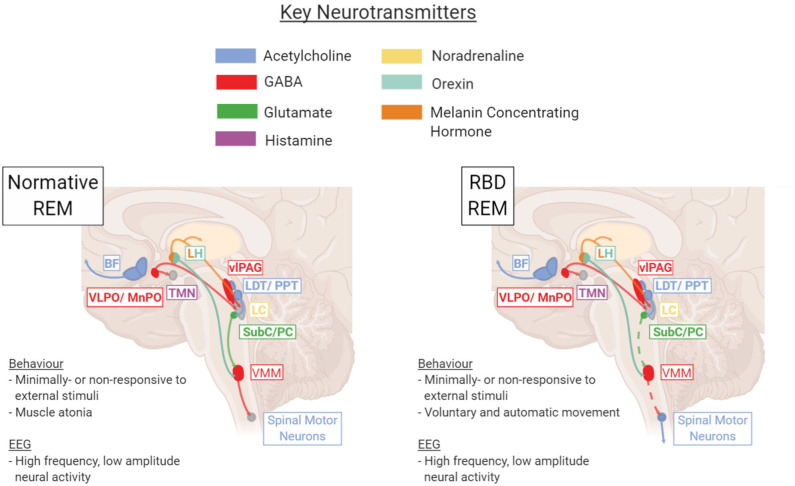
Key brain regions and neurotransmitters involved in regulation and maintenance of the REM sleep stage under healthy normative or pathological RBD conditions. In RBD, dysfunction within the SubC → VMM → Spinal Motor Neuron pathway results in a lack of REM atonia (depicted with dotted line). BF, basal forebrain; LC, locus coeruleus; LDT/PPT, laterodorsal tegmentum/pedunculopontine tegmentum; LH, lateral hypothalamus; Subc/PC, subcoeruleus/pre-locus coeruleus; TMN, tuberomammillary nucleus; vlPAG, ventrolateral periaqueductal gray; VLPO/MnPO, ventrolateral preoptic nucleus/median preoptic nucleus; VMM, ventromedial medulla. Figure created using BioRender.com.

Disruption to this process results in abnormal motor behaviors during REM sleep (Figure 1).

It is not definitively known whether RBD is caused by an imbalance originating in the glutamatergic SubC/PC or downstream in the GABA/Glycinergic VMM, though evidence from animal studies suggest the latter is more likely (14). This brainstem pathology does not exist in isolation. Given that RBD is characterized not just by an increase in small sleep twitches but also complex movements and dream enactment, it is likely that abnormal disinhibition occurs in the pyramidal motor tract during REM sleep, leading to execution of the complex movements “imagined” by the motor cortex. Imaging studies have shown that RBD can also be accompanied by changes in multiple neurotransmitter systems, including the cholinergic, noradrenergic, and dopaminergic circuits (15). Thus, one of the key challenges in treating RBD derives from the uncertainty surrounding its causative pathology and the extent of dysfunction throughout the brain.

RBD may present on its own, often referred to as idiopathic RBD (iRBD), or may exist as a secondary entity in the context another condition. Regardless of cause, all RBD subtypes are likely to reflect dysfunction at some point in the complex, interconnected REM atonia circuits.

#### “Idiopathic” RBD

These patients usually present to sleep clinics with a history of dream enacting behaviors and a present complaint of recent sleep-related injury to themselves or their bedpartner, despite no other health complaints or recent medication changes. Whilst previously considered an idiopathic phenomenon, the unquestionable link with alpha-synucleinopathies has challenged this view.

Approximately 10 years after the first description of RBD in the scientific literature, Schenck et al. reported the development of parkinsonism in ~40% iRBD individuals (16). Since then, RBD has emerged as one of the most specific predictors of the synuclein-mediated neurodegenerative diseases: Parkinson's disease (PD), Multiple System Atrophy (MSA) and Dementia with Lewy Bodies (DLB). It is now estimated that up to 90% of patients with “iRBD” will eventually develop one of the α-synucleinopathies (17).

Given that RBD is found to occur, on average, 8 years before the presentation of the core motor or cognitive symptoms required for the clinically diagnosis of PD or DLB (18), there is increasing evidence to suggest that in most cases RBD is the early manifestation, or prodrome, of a clinically-defined neurodegenerative disease. Indeed, detailed assessments often reveal subtle features of these conditions, such as hyposmia, constipation, or a slight tremor (19), that can often be missed during a clinical consultation.

Whether any truly idiopathic RBD cases exist, or whether all cases of iRBD will eventually convert to an α-synucleinopathy given sufficiently follow-up time, is currently unknown. However, given currently available evidence many in the field have moved away from the idiopathic label (3).

#### Secondary RBD

The onset of RBD symptoms may coincide with the initiation of certain drugs (20), the most common being the anti-depressant SSRIs (21) which cause RBD behaviors in up to 6% of users (22). Treatment using SSRIs results in increased serotonergic tone during both wakefulness and sleep, which in turn may interfere with mechanisms of REM atonia (21).

Other examples of secondary RBD include those caused by neurological lesion affecting sleep/wake regulatory brain regions, most commonly within the brainstem. RBD due to lesions is rare and most commonly associated with meningiomas and subsequent disruption of pontine REM-atonia structures (23), although cases of narcolepsy (24), pontine cavernoma (25), pontine lymphoma (26), multiple sclerosis (27), and acute inflammatory rhombencephalitis (28) all give further examples of RBD incidence secondary to lesion.

Though RBD may precede the diagnosis of a clinically-defined neurodegenerative disease, as seen in the majority of “idiopathic” RBD patients, it often emerges concomitantly around the same time or subsequent to a synucleinopathy diagnosis. The focus on RBD as a prodrome often leaves concomitant RBD, or RBD secondary to neurodegeneration, to fall by the wayside, with the distinction and prevalence of such cases in the α-synucleinopathy populations seldom reported in the literature.

RBD occurs concomitantly in up to 40% of PD patients (29), with studies suggesting the majority of PD patients develop RBD alongside or after their first parkinsonian symptoms (30, 31). These individuals also exhibit a more advanced disease profile (30), with greater cognitive impairment (31) compared to those whose RBD preceded their PD, warranting further investigation into the temporal spread of neurodegeneration in α-synucleinopathies. For DLB and MSA, prevalence of concomitant RBD may be as high as 76% (32) and 88% (33), respectively, though no research has investigated the timing of RBD occurrence in these populations.

The temporal variation in RBD occurrence highlights the importance of differentiating between RBD as a prodrome and as a concomitant symptom of α-synucleinopathies, especially when conducting research to phenotype and stratify RBD in α-synucleinopathic populations.

## Epidemiology

RBD typically presents from the 6th decade of life onwards, with cases of medication- or lesion-induced disease more commonly seen in those under 50 years (34).

The true prevalence of RBD in the general population is very difficult to gauge. Due to its REM state-specific occurrence, individuals are often unaware of their behaviors. Therefore, clinic-based estimates typically only capture those prompted to seek medical advice by their bedpartner or, less frequently, those that have injured themselves during violent dream enactment. Several studies have attempted to assess the prevalence in the general population using screening questionnaires, finding the rate of probable RBD to be 0.4–5% (35–37). These investigations are not without their limitations—several conditions have similar symptomology to RBD, including the increasingly common condition obstructive sleep apnoea (38), and the aforementioned prevalence rates are likely to include individuals without RBD. Polysomnography screening of the general population gives a more accurate RBD prevalence of ~1–2% (39–41).

Divergence between clinical and general population representations of RBD are further demonstrated by reported RBD sex differences. RBD is commonly regarded as a strongly male-predominant disease, largely based on clinical cohorts reporting a male to female ratio of 9:1 (42). This does not reflect population-based studies, where an equal sex split is reported (39). While sex differentials in disease can be due to true pathological mechanisms, or persist due to gender-biased underreporting, it is unknown which accounts for the sex difference seen in RBD. It has been speculated that men are naturally more aggressive than women and therefore are more likely to experience violent dreams and RBD behaviors. However, studies have shown that RBD dream content does not differ between sexes (43, 44) and violent dreams are not associated with higher testosterone levels (45). If and why women are susceptible to underreporting RBD, as in the case of snoring and obstructive sleep apnoea (46, 47), therefore needs further investigation. Interestingly, male sex is an identified risk factor for all of the α-synucleinopathies (48–51), though the reason for this remains unknown.

As well as male sex and antidepressant use, large cross-sectional cohort studies have consistently identified low socioeconomic status and jobs with toxic environmental exposures (e.g., farming or mining) as environmental risk factors for RBD development (35, 36, 52). Additional measures associated with compromised health—including smoking, drinking, low physical activity, cardiovascular risk factors (such as diabetes), and psychological distress—have also been implicated (35, 36, 52). The complex interplay between most of these factors is not specific to RBD and rather reflects the influence of social structures upon population health. Interestingly, the link between occupational and environmental exposures (most notably pesticides) and RBD is one that mirrors the PD population. The link between pesticides and MSA or DLB is less clear (50, 53, 54), suggesting alternative triggers for the specific strain pathology of these conditions.

RBD is non-familial and therefore susceptibility to disease development is likely to be a combination of multiple environmental and genetic determinants. Studies of RBD genetics tend to center upon single nucleotide polymorphisms (SNPs) and genetic mutations known to be associated with the α-synucleinopathies. Genetic changes in RBD populations include underrepresentation of a PD-protective MAPT (Microtubule Associated Protein Tau) SNP (55), glucocerebrosidase missense variant overrepresentation (56), altered clock gene expression (57), and SNCA (Synuclein Alpha) gene variants (58). In each instance, however, the mutations are present in only a small minority of the RBD population thus limiting the conclusions that can be drawn regarding genetic determinants of RBD.

### Patient Presentation and Diagnosis

Of those that seek medical advice, patients will usually present to their GP in the instance of self- or bedpartner injury due to their dream-enacting behaviors. In the authors' experience, this is often a significant barrier to accessing help, with many patients, and GPs, not recognizing RBD as a medical problem. This is reflected by the diagnostic delay seen in RBD, cited between 7 and 9 years on average (59, 60).

When RBD is suspected outside of the setting of a designated sleep clinic, simple screening questionnaires, such as the single-question RBD1Q (61), or the more detailed RBDSQ (62), may be used to prompt further assessment or onward referral.

Whilst these questionnaires hold some value when screening for the disorder, they do not inherently encompass the diagnostic criteria, and the cut-off points used are somewhat contentious (63). Thus, these scales are yet to be used as a standardized clinical resource.

**Figure 2:**
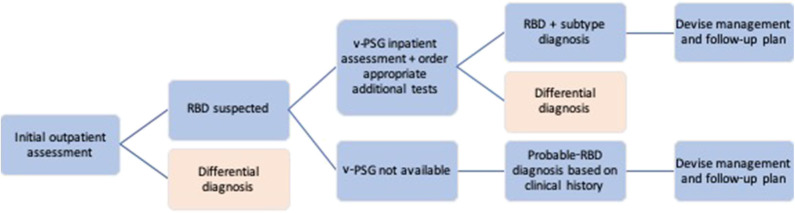
Flow chart summarizing the process of RBD diagnosis.

Individuals suspected of having RBD should be referred to a specialist Sleep Medicine or Neurology service for a diagnostic assessment (see Figure 2 for an outline of the RBD diagnostic process). Although there is likely significant variability between the individual clinicians (as there are no ICSD3 or other guidelines for RBD diagnostic assessment), this initial outpatient clinical assessment broadly consists of a general examination and neurological examination to rule out differential diagnoses, coupled with several tests, and rating scales designed to assess more specific sleep and neurodegenerative aspects of the condition. Whenever possible, clinicians should make use of any information from bed-partners, as they often provide a more accurate history of sleep-related behaviors. These are also the most reliable means to assess the severity of the RBD, which, in turn, can aid management options. Clinicians should additionally take detailed note of history indicative of secondary RBD, as well as screen for α-synucleinopathic prodromal symptoms such as hyposmia, constipation, and cognitive changes.

Where indicated from the history and clinical examination, further investigations e.g., brain MRI, may be required to diagnose secondary RBD and inform treatment. The utility of DaTSCAN for diagnosing RBD secondary to α-synucleinopathy is often limited at this stage and is not recommended (see “Current Research: RBD and the α-Synucleinopathies” section for further discussion).

The gold-standard protocol for RBD diagnosis is a clinical assessment coupled with a subsequent overnight video polysomnography (v-PSG) study. The collective measures from these form the basis of the most commonly used diagnostic criteria, the International Classification of Sleep Disorders (3rd Edition) (ICSD-3) (see Table 1).

**Table 1:** Diagnostic criteria for REM sleep behavior disorder (4).

**Criteria**	**Description**
1	Repeated episodes of sleep-related vocalization and/or complex motor behaviors
2	These behaviors are documented by polysomnography to occur during REM sleep or, based on clinical history of dream enactment, are presumed to occur during REM sleep
3	Polysomnographic recording demonstrates REM sleep without atonia (RSWA)
4	The disturbance is not better explained by another sleep disorder, mental disorder, medication or substance abuse

*All 4 criteria must be met for a definite RBD diagnosis. If v-PSG is not available, or RSWA is not captured during v-PSG then a provisional diagnosis of RBD can be given if all other criteria are met*.

While the diagnosis of probable RBD can be solely made based on a detailed history, meeting points 1, 2, and 4 on the ICSD-3 RBD Diagnostic Criteria, a confirmatory v-PSG study is required for a formal diagnosis to be made. The v-PSG is an inpatient sleep study, and therefore may require the patient to be further referred to a specialist sleep center. The study itself consists of electroencephalography (EEG) to assess brain activity, electromyography (EMG) for muscle activity, respiratory, oximetry, and heart rate monitoring and video recording of the patient while they sleep. It is also advisable for the patient to undergo a next-day Multiple Sleep Latency Test (MSLT) to control for excessive daytime sleepiness and narcolepsy.

The minimum aim of the v-PSG is to capture RSWA, which the ICSD-3 states is indicated by “excessive augmentation of chin EMG” or “excessive chin or limb phasic EMG twitching” during REM sleep (4). Scoring guidelines from the American Academy of Sleep Science (AASM) quantitatively define these as “a chin EMG amplitude greater than the minimum amplitude demonstrated in NREM sleep” and “transient muscle activity 0.1–5.0 s in duration at least 4 times as high amplitude as background EMG,” respectively (64). Such twitches occur relatively frequently in RBD individuals so the likelihood of capturing RSWA during a diagnostic v-PSG is high. It is the large dream enactments and complex motor behaviors which are less common (for instance, which an individual may only experience once a month) and therefore rarer to capture during the sleep study. Therefore, the diagnostic criteria allow for RBD to be diagnosed based on v-PSG-confirmed RSWA with a *history* of dream enactment or sleep behaviors, acknowledging the limitations of a one-night sleep study for full phenotype capture.

The v-PSG requires not only a sleep center to physically host the study, but also technicians to score and interpret the data. The resources available to a health service will therefore determine access to v-PSG.

Though seemingly an attractive option given the limited access to sleep studies in most centers, the practice of solely relying on clinical assessment or screening questionnaires is likely to result in a significant number of false-positive diagnoses (65). This is particularly pertinent in suspected idiopathic cases, where a diagnosis of RBD should prompt discussion regarding future risk of developing a neurodegenerative disorder (see “RBD Prognosis and Communicating the Risks” section).

## RBD Prognosis and Communicating the Risks

The prognosis for RBD depends largely upon the subtype. Patients diagnosed with RBD secondary to medication have the most promising prognosis of RBD resolution once the causative medication is withdrawn. However, it has been shown that RBD may persist following cessation of SSRIs (66, 67), and it is therefore possible that in some cases the medication simply “unmasked” an already underlying pathology, triggering early clinical presentation (22). For RBD secondary to defined lesion e.g., inflammatory plaques, the main symptoms of RBD can be controlled relatively reliably using a combination of pharmacological and behavioral treatments. As these patient's present with chronic but stable neural tissue damage, their RBD symptoms are unlikely to change over time.

For patients with RBD presenting as part of a clinically-defined neurodegenerative condition, such as PD, MSA, or DLB, the management of their sleep disorder should form part of their holistic care. Generally, the presence of RBD marks a less-favorable disease phenotype. In PD, for example, the presence of concomitant RBD is associated with a greater non-motor burden and a more adverse prognosis (68–71). There have been no studies of whether the symptomatic treatment of RBD impacts long-term outcomes in these patients.

Finally, for patients diagnosed with apparently idiopathic disease, the prognosis remains uncertain. There are currently no biomarkers or investigations to determine the personal risk of developing an α-synucleinopathy. A recent metanalysis of the existing international prospective cohort studies found that after an average follow up of 4.6 years, 352 (28%) of 1,280 RBD patients were diagnosed with a clinically defined neurodegenerative disorder. Of those, 52% developed PD, 43.5% developed DLB, and 4.5% developed MSA (18). Color vision deficits, hyposmia, erectile dysfunction and constipation accompany the loss of REM sleep atonia in the “prodromal” disease stages, before a cluster of additional symptoms including cognitive deficits, urinary dysfunction and motor symptoms arise in the “preclinical” stage, defined as <5 years before diagnosable phenoconversion (72). Importantly, severity of all prodromal and preclinical symptoms increases over time (72), reinforcing the onus on clinicians to undertake vigilant symptom tracking of RBD patients to inform patient management and scientific research.

It is the responsibility of the clinician making the diagnosis to sensitively and clearly communicate the risks of neurodegeneration associated with RBD to their patients. Although there are no official UK guidelines for the care of RBD patients, it is in the patient's best interest to be fully informed of their condition (73). Not only does this respect and maintain the autonomy of the patient, it also facilitates the conversation of current RBD research and their potential involvement (74). It is highly beneficial for the RBD and α-synucleinopathy research fields if all patients diagnosed with RBD, regardless of subtype but especially those with iRBD, are encouraged to participate in experimental research. Further to any clinician-patient conversations, patient counseling, and advice services should be made available and recommended to the individual.

## RBD Management

Given the general uncertainty of causative pathology and prognosis for RBD, the two greatest challenges for clinicians remain the successful management of the condition, and in the case of idiopathic or suspected prodromal RBD, symptom-tracking for neurodegeneration indicators. An overview of RBD patient management is provided in Table 2.

**Table 2:** Summary of RBD patient management recommendations according to condition subtype.

**RBD subtype**		**Recommendation**			**Patient follow-up**
		**First-line**	**Second-line**	**Third-line**	
Idiopathic	iRBD	Behavioral	Clonazepam 0.25–0.5 mg or melatonin modified-release 2 mg according to symptom severity	Clonazepam 0.25–0.5 mg or melatonin modified-release 2 mg	Regular 6 month-1 year follow-up. Review of current treatments, dosage titration if appropriate and monitoring of any motor, physiological, or cognitive changes
Secondary	Medication	Cessation and replacement of causative medication or dosage titration to stop RBD symptoms	Behavioral	Check medication contraindication and proceed with prescription of clonazepam 0.25–0.5 mg or melatonin modified-release 2 mg	Initial short-term follow up for review of treatment and dosage titration if appropriate. Further review at patient's request
	Lesion	Behavioral	Clonazepam 0.5 mg or melatonin modified-release 2 mg according to symptom severity	Clonazepam 0.25–0.5 mg or melatonin modified-release 2 mg	Initial short-term follow up for review of treatment and dosage titration if appropriate. Further review at patient's request
	α-synucleinopathy	Behavioral	Clonazepam 0.25–0.5 mg or melatonin modified-release 2 mg according to symptom severity	Clonazepam 0.25–0.5 mg or melatonin modified-release 2 mg	Initial short-term follow up for review of treatment and dosage titration if appropriate. Further review at patient's request

*Titration of clonazepam dosage should be within the ranges of 0.25 mg−2.0 mg/night. Doses of modified-release melatonin are in the range of 2–6 mg, with higher doses up to 12 mg occasionally used*.

## Treatment

The treatment of RBD falls into two categories: pharmacological and behavioral. Unfortunately, as no cure for the disorder exists, management remains symptomatic, with highest priority placed on controlling the extreme and potentially injurious motor behaviors. Many patients will, therefore, elect not to pursue any treatment, especially when the impact of the condition on their quality of life is low.

## Behavioral

As there are no reported associations between daytime events (e.g., stress, alcohol intake) and subsequent night-time RBD behaviors (9), behavioral recommendations focus on the creation of a safe sleep-environment. This can include removing or padding bedside furniture, or lowering the mattress and placing pillows on the floor beside the bed in case of falls (75). In some cases, it may be recommended for the patient and bedpartner to sleep in separate beds to minimize injury. Behavioral recommendations are applicable to both idiopathic and secondary RBD patients, and the extent of these measures should be appropriate to the severity and nature of the patient's RBD symptoms.

Besides changing the immediate sleep environment, physicians should as always encourage patients to observe good sleep hygiene and a healthy lifestyle. The lack of unique risk factors for RBD will however limit the specificity, and likely the efficacy, of this advice.

## Pharmacological

Clonazepam is the generally the first-line agent used for the treatment of RBD symptoms. It is long-acting benzodiazepine with a half-life of 30–40 h, and typically commenced at a starting dose of 0.25–1 mg, taken nightly at bedtime. Clonazepam acts to non-specifically enhance inhibitory processes within the brain by binding to GABA_A_ receptors, and thus temporarily quells overactive or disinhibited brain regions which control REM atonia, ultimately reducing the number of motor behaviors during subsequent sleep. It was first explored as a treatment for RBD given its efficacy in treating periodic leg movement disorder (76), another sleep condition characterized by excessive motor behaviors.

There have been no randomized, double-blind, controlled trials of clonazepam in an iRBD population, and only a handful of studies have looked at the effects of the drug on sleep and RBD symptoms. Clonazepam somewhat restores the EEG spectral profile of iRBD individuals to that of controls when compared to their drug-naïve counterparts, as well as improving both NREM and REM sleep stage stability (77, 78). However, long-term clonazepam use did not affect subjective measures such as daytime sleepiness (77) nor affect the REM sleep atonia index (77, 78). Neither study captured complex, violent REM movements in either the drug-naïve or clonazepam-treated iRBD groups and thus no conclusion can be drawn on the efficacy of clonazepam in reducing the severity of complex movements. However, if judged on RSWA index alone, clonazepam use does not sufficiently control muscle tone during RBD sleep.

Naturalistic follow-up studies have found similar results to the above cross-sectional studies. Li et al.'s v-PSG follow-up study found long-term clonazepam use increased the amount of stage 2 NREM sleep but failed to reduce the amount of REM sleep atonia in iRBD individuals; in fact, the amount of total and tonic RSWA significantly increased over time despite clonazepam use (79), demonstrating the progressive nature of the condition. In a follow-up survey, the majority of iRBD patients receiving clonazepam continued to experience sleep behaviors despite treatment, though frequency, severity and number of dream enactment behaviors were all reduced (80).

Over time, a number of patients will stop taking clonazepam due to side effects, while many of those who remain on clonazepam tend to experience the emergence of residual RBD symptoms (79).

The above follow-up study outcomes are relatively standard for long-term benzodiazepine use which, while a clinical common practice (81), should in and of itself be carefully considered case-by-case. Long-term benzodiazepine use is generally defined as ≥6 months (81) and holds the greatest risk for adverse effects such as cognitive impairment (82), dementia development (83), and risk of falling (84), in aged populations. While long-term benzodiazepine use in the elderly is generally maintained at a stable, albeit higher than average, dosage (81) it is common for clonazepam doses progressively over time in the RBD population (79, 85). Underlying this are three potential causes—dosage titration, the development of a tolerance to clonazepam or a progressive worsening of RBD symptoms over time. Long-term assessment of RBD symptoms does indeed show the latter (79, 86), and in light of conflicting evidence for long-term clonazepam tolerance in chronic conditions (87–89) it should generally be assumed that clonazepam dosage should be monitored closely to ensure sufficient control of RBD symptoms.

Clonazepam must be used with caution in the elderly, individuals with a history of depression and those with airways obstruction (90), such as obstructive sleep apnoea which is commonly concomitant with RBD (91). Additionally, the long half-life of clonazepam (92) can lead to “hangover” side effects of excessive daytime sleepiness the next morning (79). Efforts to replace or complement clonazepam therapy in unresponsive or non-tolerant patients has led to the exploratory prescription of alternative drugs with some success, namely in the prescription of zopiclone (93), sodium oxybate (93), or pramipexole (94) [for further review, see (95)]. As these cases are limited in size and are not extensive case-controlled studies, clonazepam remains the chosen pharmacological treatment for RBD, if only for the sole reason of upholding the status quo.

Over recent years melatonin and associated melatonergic agents have established their place in the management of RBD. Melatonin is indicated for the treatment of chronobiological disorders and insomnia, though is often prescribed off-label for all other sleep disorders. One of melatonin's main functions is to synchronize circadian rhythms by binding to its receptors at the hypothalamic suprachiasmatic nucleus (SCN) (96). Melatonin has been shown to improve sleep quality and duration in both healthy and diseased individuals (97, 98), and as sleep disorders are often multi-factorial, prescribing melatonin or melatonin-related compounds is often done to try to non-specifically stabilize any underlying circadian desynchronizations. In the case of RBD, where changes in REM circadian rhythmicity have been shown (57, 99), blanket-prescription of melatonin may be beneficial for patients. However, as no blinded case-controlled trials have confirmed the stabilization of REM circadian rhythmicity by melatonin in RBD, the clinical- and cost-effectiveness of blanket prescription policy for healthcare systems should be taken into consideration by the clinician (100).

Enhancement of melatonin signaling within the brain can be achieved directly with an exogenous, modified release form of melatonin or indirectly with the melatonin receptor agonist Ramelteon. Melatonin is usually prescribed to treat RBD behavioral symptoms in the context of clonazepam shortcomings—either as a replacement monotherapy (if the patient cannot tolerate clonazepam due to side effects) or as a polytherapy in combination with clonazepam (if clonazepam is insufficient in controlling RBD symptoms and residual sleep behaviors persist).

The evidence for melatonin's efficacy is variable: several studies have found it to reduce RBD motor behavior occurrence (101–103), with long-term use ameliorating RBD symptoms in the majority of patients (104). However, a recent placebo-controlled trial found melatonin use at either 2 or 6 mg/night improved self-reported measures such as daytime sleepiness, sleep quality or dream enactment behaviors (105). A recent randomized, controlled trial of melatonin in a cohort of PD patients with RBD also found no effect of melatonin on RBD symptom frequency or severity (106), suggesting melatonin may be less effective at controlling RBD symptoms in the context of advanced neurodegeneration. The efficacy of melatonergic compounds such as the melatonin receptor agonist Ramelteon also do not significantly improve RBD symptom severity (107).

As melatonin causes fewer side effects has low tolerance risk and few drug interactions, it may be more suitable for RBD patients than clonazepam (108). Despite this, the uncertainty around melatonin's mechanism of action and overall efficacy means the popularity of clonazepam prevails.

At the time of writing, the UK RAG drug classification lists melatonin as a RED drug and clonazepam as a GREEN drug. The prescription of melatonin therefore requires secondary or tertiary care initiation and management whereas clonazepam prescription can be managed by GPs. This may be a choice-limiting factor for some patients. Ultimately, it is a combination of the clinician's personal preference and best judgement of which drug is prescribed.

## Symptom Monitoring

Follow-up studies and projected conversion rates estimate that the majority of idiopathic RBD patients will develop a clinically-defined α-synucleinopathy within 8 years of their initial diagnosis (18). Despite this, there are no guidelines for the routine monitoring of RBD patient symptomology (95). What further limits any attempts at symptom tracking is the lack of standardized clinical rating scales for RBD severity progression and conversion. The discrepancy between a lack of clinician-instigated patient follow up vs. the high risk of α-synucleinopathy development is largely explained by the fact that there are no medical interventions to stop or slow RBD conversion. The benefits of tracking neurodegenerative symptoms are therefore greatly outweighed by the economic cost to the healthcare system and the emotional burden to the patient.

The immediacy of such a situation, wherein both clinician and patient are powerless, can make symptom monitoring unattractive. Therefore, to date, the majority such follow up tends to be confined to research studies. Below, we detail how current practice in RBD management could be feasibly improved without changes to existing healthcare practices or clinic frameworks and explore possibilities for the development of a prodromal rating scale to track idiopathic RBD symptomology.

Ideally, once RBD has been confirmed on v-PSG, the newly diagnosed patient should undergo a series of standardized functional assessments to determine their current or “baseline” symptoms. These assessments should address the range of deficits which have been associated with RBD (and subsequent α-synucleinopathy development) in the literature and should be sensitive enough to capture subtle dysfunctions. Ultimately, the development of a unified assessment scale for clinical-practice deployment to RBD patients is recommended. This should be reflective of the Movement Disorders Society's recently published research criteria for prodromal PD (109), which demonstrates relatively high sensitivity for prediction of PD development (110). RBD patients should then be seen annually to discuss their disease phenotype with their clinician, and to repeat the functional assessments. This would generate an in-depth profile of each patient and their symptoms and ensure early signs of neurodegeneration can be addressed using available clinical tools.

## Current Research: RBD and the α-Synucleinopathies

The tests described in this section are used solely in experimental settings and therefore clinicians are not able to use them in the clinical RBD diagnostic or prognostic process. The majority of RBD research focuses upon the relationship between RBD and subsequent α-synucleinopathy development. In particular, the search for biomarkers which identify underlying α-synuclein pathology and predict RBD phenoconversion is perhaps the most relevant for clinical practice. As discussed previously there are no genetic markers with predictive power for RBD phenoconversion, and while rating scales may identify individuals with a high risk of phenoconversion, they do not have a binary outcome measure- a feature which is essential in prognostic testing.

Biomarkers for RBD conversion are essentially testing whether the RBD features of an individual are due to underlying α-synuclein pathology or not. This can be done by testing for misfolded, pathological α-synuclein (typically identified by serine-129 phosphorylation) in peripheral tissues and fluid samples. Dermal nerve fibers (111, 112), cerebrospinal fluid (113), submandibular glands (114), colonic submucosal nerve fibers (115), salivary glands (116), and parotid glands (117) have all been found to contain pathological α-synuclein proteins in idiopathic RBD patient populations and confirmatory PD populations. Such investigations are in their infancy but show promise as a basis for relatively low-cost clinical diagnostic biopsy tests for alpha-synucleinopathy in RBD patients.

Bioimaging techniques, such as MRI and PET scanning, are a less invasive alternative to biopsy-based diagnostic tests. Such methods are capable of identifying and quantifying dysfunction in deep structures which may otherwise be inaccessible. In general, the greatest focus has been upon tissues and neurotransmitters whose dysfunction corresponds to the symptoms of α-synucleinopathies as there are no tracer molecules for direct pathological α-synuclein visualization. The degeneration of dopaminergic neurons seen in PD, and to some extent DLB, has led to an emphasis on imaging dopamine-associated molecules, such as dopamine and non-specific monoamine transporters, in RBD patients (118). DaTSCANs, which are a way to visualize presynaptic dopamine transporter density, are already used clinically in the diagnosis of PD and may also be enlisted in confirming whether an RBD individual has degeneration of dopaminergic terminals projecting to the putamen. However, RBD onset likely precedes gross dopamine dysfunction, and this is reflected in the literature that not all RBD patients present with abnormal DaTSCAN (18). Efforts to image neurotransmitter systems which may be affected earlier in the prodromal period accompanying RBD symptoms are therefore underway. Perhaps the most thorough and ambitious of these efforts demonstrated multimodal characterization of the sympathetic, parasympathetic, noradrenergic, and dopaminergic systems in RBD patients, using a combination of MRI, PET, CT, and scintigraphy (15). The authors found that the RBD patients had abnormal peripheral autonomic nervous system and brainstem results, but few demonstrated cerebral pathology (15). These results are in line with both clinical DaTSCAN findings and the Braak staging model of pathology and argue against the use of DaTSCAN for RBD diagnosis or prognosis prediction.

Though providing a useful insight into the progression and extent of neurodegeneration in an individual, there are several caveats to the use of imaging tests in regular diagnostic or prognostic practice. As seen above, DaTSCANs are unlikely to yield valuable results in an RBD patient, making the tests highly uneconomical. Multiple imaging tests are also inherently accompanied by radiation exposure and its associated risks. Therefore, while there is potential for assessment of alternative neurotransmitter systems in RBD patients, the clinical value of such tests must be assessed further. As with the majority of tests, neuroimaging currently adds to the overall picture rather than produces a binary diagnostic or prognostic outcome.

## α-Synucleinopathy Treatments

Advances in preventative, slowing or curative α-synucleinopathy treatments would add credence to RBD patient monitoring, as the potential for therapeutic deployment during the RBD-characterized prodromal period could significantly disrupt the disease trajectory. Development of antibodies against pathological α-synuclein (119, 120) and neurorestorative/neuroprotective compounds (121) hold promise for treatments but as yet there are no clinically-approved α-synucleinopathy therapies.

## Conclusions

RBD presents a multitude of considerations relevant for neurologists and non-specialized clinicians alike. From a public health perspective, RBD highlights the importance of sleep for good health and the need for greater awareness of sleep disorders and their detrimental effects. For scientists and researchers, RBD represents a window of opportunity for deployment of new interventions against neurodegenerative processes, as well as an opportunity to gain insight into the complex neural mechanisms of sleep and wake. Finally, the diagnosis, treatment, and ethical considerations of RBD require the clinician to demonstrate cross-speciality knowledge, emphasizing the importance and challenges of sleep medicine training in an over-stretched education system which provides inadequate training on sleep and it's disorders (122–124).

The dream enactment characteristic of RBD is associated with a variety of root causes- from acute emotional states to progressive neurodegeneration. The biological mechanisms underpinning the intrusion of waking behaviors into REM sleep remain to be fully characterized, plus it remains to be seen whether the same mechanisms underlie different disorders which share the symptom of dream enactment. For the majority of RBD patients, dream enactment behaviors will be the first symptom of impending α-synucleinopathic disease. Thus, the onus lies with the clinician to recognize these risks, communicate them effectively, and diagnose accordingly. While the prognosis of idiopathic RBD remains uncertain, an increase in basic and clinical research into the condition is already leading to greater understanding and endpoint prediction. The final barrier to RBD patient care remains effective treatments to slow, reverse, or stop the effects of α-synuclein mediated disease.

## Author Contributions

MR and AR conceived of the presented idea. AR drafted the manuscript with support from DR, AW, MJ, and MR. All authors contributed to the final version of the manuscript.

## Conflict of Interest

The authors declare that the research was conducted in the absence of any commercial or financial relationships that could be construed as a potential conflict of interest.
